# The utilization of decision trees on orthopantomographic and lateral panoramic graphs for the diagnosis of unilateral anterior disc displacement of the temporomandibular joint

**DOI:** 10.1186/s12903-024-04121-z

**Published:** 2024-03-16

**Authors:** Muhammed Enes Naralan, Binali Cakir, Kaan Orhan

**Affiliations:** 1 Department of Dentomaxillofacial Radiology, Faculty of Dentistry, Recep Tayyip Erdoğan University, Rize, 53020 Turkey; 2 Department of Dentomaxillofacial Radiology, Faculty of Dentistry, Atatürk University, Erzurum, Turkey; 3 Department of Dentomaxillofacial Radiology, Faculty of Dentistry, Ankara University, Ankara, Turkey

**Keywords:** Temporomandibular joint, Anterior disc displacement, Magnetic resonance imaging, Orthopantomography, Lateral panoramic graphy

## Abstract

**Background:**

Investigation is to utilize decision trees in conjunction with orthopantomography (OPT) and lateral panoramic graphy (LPG) to diagnose unilateral anterior disc displacement (ADD) of the temporomandibular joint.

**Methods:**

In this study, 161 patients with images obtained through all three imaging methods, MRI, OPT, and LPG, were selected from the archives. The participants were categorized into two groups: the study group, comprising 89 patients with unilateral anterior disc displacement, and the control group, consisting of 72 healthy individuals. Measurements, including 2 angles (antero-posterior angle and superior-inferior angle) and 3 distance parameters (anterior joint space distance, superior joint space distance, and posterior joint space distance), were conducted on each imaging modality dataset. To assess the obtained measurement data within each patient, the differences from each measurement were calculated. Statistical analysis of the measurement differences between the control and study groups was carried out with independent t test, and decision trees were generated using the SPSS 25 decision tree module 5.0.

**Results:**

In ADD patients, it was statistically significantly found that the APA increased while the SIA decreased for angle measurements. But for linear measurements, AS increased while the SS and PS decreased in MRI, OPT, and LPG.

**Conclusion:**

ADD can be diagnosed in OPT and LPG. The identification of the specific type of ADD that occurs in the temporomandibular joint is not feasible.

## Introduction

Musculoskeletal disorders of the temporomandibular joint (TMJ) can arise from physical or psychological factors or a combination of both. Accurate diagnosis and appropriate treatment are crucial, as TMJ pathologies have a universal impact on individuals of diverse ages and genders. Anterior disc displacement (ADD) is a pathological condition that impacts both the soft and hard tissues. Given that the ailment in question pertains to soft tissue, the optimal approach for diagnosis would be through the employment of magnetic resonance imaging (MRI). MRI provides a detailed visualization of both hard and soft tissues within the TMJ structure. The utilization of MRI in TMJ imaging is hindered by the challenges of accessibility and cost, as well as the need for specialized expertise in image interpretation. Moreover, the limited availability of MRI facilities results in a prolonged duration for conducting diagnostic procedures.

Orthopantomography (OPT) devices can acquire lateral panoramic radiography (LPG) for the purpose of imaging the TMJ. OPT and LPG is viable options for TMJ imaging. The prevalent utilization of OPT and LPG for the assessment of the TMJ is noteworthy in the context of routine dental procedures. The broad imaging scope of OPT holds significant value in the identification and assessment of various medical conditions. Nevertheless, the existing literature presents substantial evidence indicating that the assessment of TMJ through OPT is inadequate [1, 2]. The ease of interpretation of OPT and LPG surpasses that of MRI, despite the latter being widely accessible and having a shorter acquisition time.

While OPT and LPG imaging techniques do not allow for the evaluation of soft tissue, they do enable the observation of the bone structures of the TMJ. Nevertheless, the oblique X-rays utilized in acquiring OPT and LPG hinder the possibility of precise joint space assessments [3–6]. Nevertheless, due to the observability of hard tissues like bones, it is possible to establish fixed anatomical landmarks and track the alterations in measurements between these landmarks [4–9].

According to existing literature, morphological asymmetries can manifest in both radiological and clinical joint structures as a consequence of TMJ disorders. An examination of these asymmetries can lead to a diagnosis, as demonstrated by previous research [10–12]. Studies have reported the occurrence of superior and posterior displacement of the condyle in individuals diagnosed with ADD, specifically in relation to TMJ. Additionally, articles have reported that the estimation of disc displacement can be achieved through the analysis of alterations in the joint space [10–14]. The findings of these studies indicate that ADD is associated with alterations in the positional alignment of the TMJ bone structures.

The objective of this investigation is to identify unilateral ADD by utilizing a decision tree constructed from the measurement discrepancies derived from anatomical landmarks in OPT and LPG on radiology archive images in a retrospective manner. The null hypothesis is that there is no significant difference in anatomic measurements between patients with ADD and healthy individuals.

## Material and methods

The present investigation was conducted in a retrospective manner, adhering fully to the relevant ethical principles, which encompassed the World Medical Association Declaration of Helsinki of 1964 and a subsequent iteration. The Ethics Committee of the Faculty of Medicine at Atatürk University approved study (approval number: 08/51).

Images obtained from the archives of Atatürk University Faculty of Medicine Department of Radiology and Atatürk University Faculty of Dentistry Department of Dentomaxillofacial Radiology between 2015 and 2018 were utilized in this study. Patients referred to radiology departments for TMJ examination were screened and a total of 467 patients were included in the study. The study analyzed patients who were specifically chosen through OPT (ProMax (Planmeca, Helsinki, Finland), LPG (ProMax (Planmeca, Helsinki, Finland), and MRI (1.5 Tesla Siemens Magnetom Avanto and 3 Tesla Siemens Magnetom Skyra (Siemens Medical Systems, Erlangen, Germany) images. Common exposure parameters were applied during image acquisition for all patients. Specifically, for OPT, the parameters were set at 66 kVp, 8 mA, and 16.2 s. Meanwhile, for LPG, the exposure settings were 66 kVp, 6.3 mA, and 16 s. The patient was positioned in centric occlusion, and an OPT-standardized bite block was utilized for positioning during the acquisition of MRI and LPG in the closed position, following standard procedure. As per the guidelines provided by the manufacturer, the Frankfurt horizontal plane was maintained parallel to the ground, while the vertical line was aligned parallel to the sagittal plane.

The MRI technique was employed to assess the TMJ of the subjects in the research cohort. The study analyzed open and closed positions using T1, T2, and proton density sequences without the administration of contrast agents. In cases where unilateral ADD is being diagnosed, a closed position is established when the articular disc surpasses the 12 o'clock position of the condyle by a margin of + 10 degrees within the oral cavity. The present study assessed cases in which the disc was observed to relocate on the condyle in images obtained with the mouth open as indicative of anterior disc displacement with reduction (ADDwR). Conversely, cases in which the disc did not relocate on the condyle were classified as anterior disc displacement without reduction (ADDwoR).

This study differentiated between two distinct patient groups: those presenting with radiological health and those exhibiting unilateral ADD, either with or without reduction. The inclusion criteria for the study group specifically involved patients with unilateral ADD, excluding individuals with conditions such as other unilateral disc displacements besides ADD, bilateral disc displacement of any type, unilateral or bilateral subluxation, cortical and/or trabecular bone irregularities from degenerative changes, systemic diseases, effusion in TMJ structures, TMJ trauma, abnormal TMJ morphology, TMJ pathologies, and instances where MRI, OPT, and LPG images were missing. Additionally, the study excluded patients with distorted radiological images deemed unsuitable for examination due to artifacts and/or technical issues, as well as those with errors in patient positioning. These rigorous inclusion and exclusion criteria were meticulously applied to ensure a focused and homogeneous study cohort, allowing for a more precise investigation into the relationships between radiological findings and the presence or absence of unilateral ADD.

The inclusion criteria for the control group included patients with radiologically healthy TMJs. Exclusion criteria for the control group included any unilateral disc displacement and exclusion criteria for the study group.

All participants were included in the study, regardless of age and gender. To reduce potential sources of bias, measurements were performed in a random and blinded manner, following group assignment determined by a single observer with expertise in oral and dentomaxillofacial radiology. To ensure this, each patient's data is anonymized. Then, all data folders were recorded collectively without being categorized and measurements were made. Then, the measurements were made again 1 month later and the intraclass correlation coefficient (ICC) was calculated, and the MRIs were re-evaluated and grouped.

**Fig. 1:**
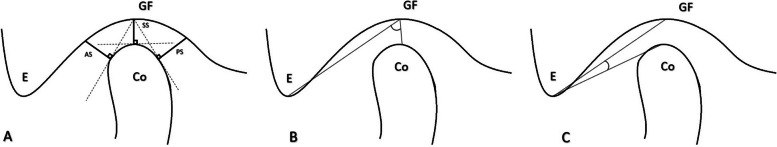
Measurements made in TMJ **A**. Distance measurements **B**. Antero-posterior Angle (APA) **C**. Supero-inferior Angle (SIA). Co: Condyle, E: Articular eminence, GF: Glenoid Fossa, AS: Anterior Space, SS: Superior Space, PS: Posterior Space

The parameters necessary for comprehending the displacement in the condyle and developing the decision tree encompassed three spatial measurements in OPT, LPG, and MRI, namely anterior space (AS), superior space (SS), and posterior space (PS), in addition to two angle measurements, namely supero-inferior angle (SIA) and antero-posterior angle (APA), each of which was ascertained using two fixed points, the glenoid fossa and articular eminence, and one variable point, the condyle (Fig. 1).

**Fig. 2:**
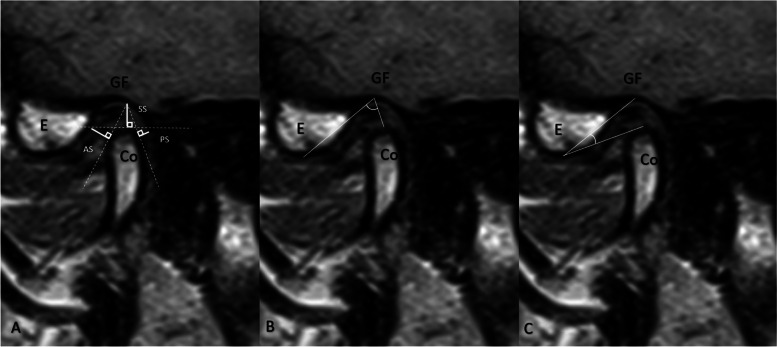
TMJ measurements in MRI. **A** Distance measurements. **B** Antero-posterior Angle (APA) **C**. Supero-inferior Angle (SIA). MRI: Magnetic resonance imaging, Co: Condyle, E: Articular eminence, GF: Glenoid Fossa, AS: Anterior Space, SS: Superior Space, PS: Posterior Space

**Fig. 3:**
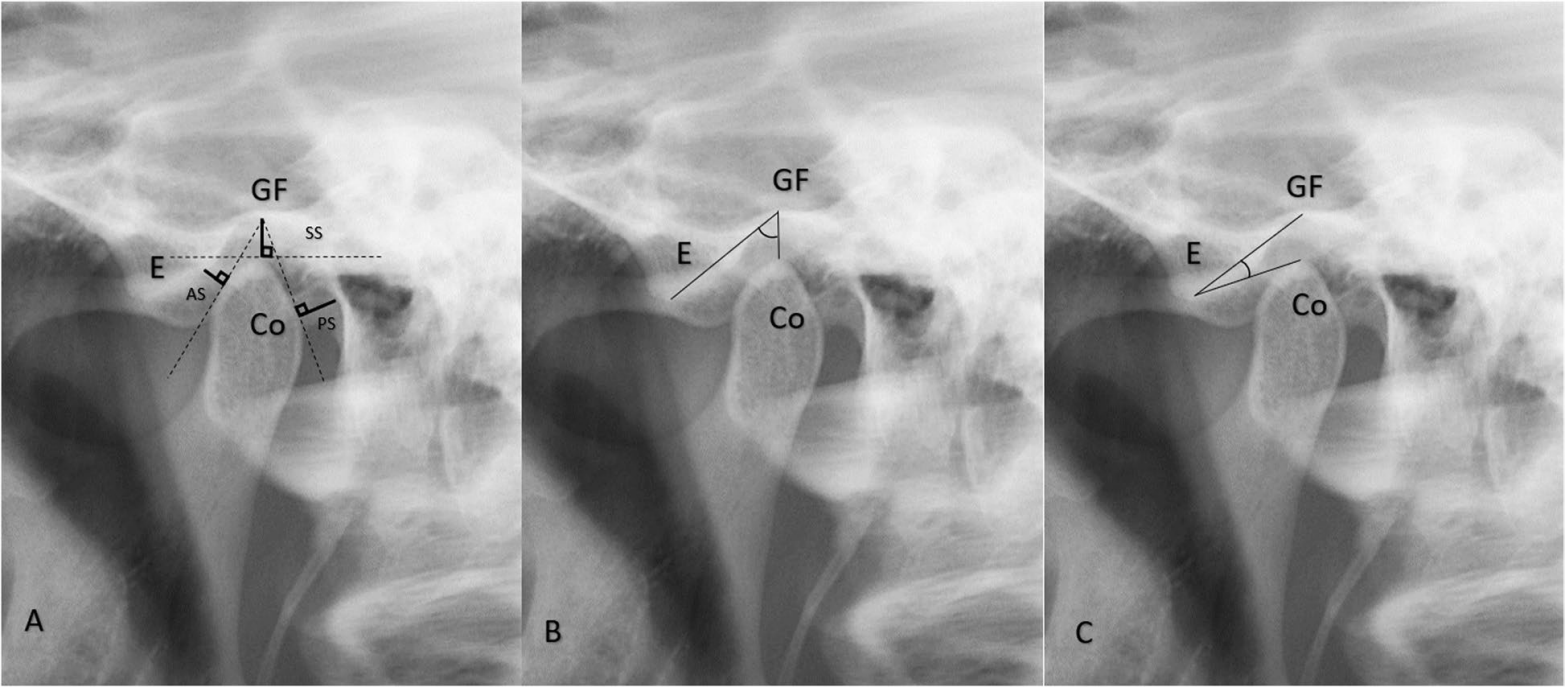
TMJ measurements in OPT. **A** Distance measurements **B**. Antero-posterior Angle (APA) **C**. Supero-inferior Angle (SIA). OPT: Orthopantomographic Co: Condyle, E: Articular eminence, GF: Glenoid Fossa, AS: Anterior Space, SS: Superior Space, PS: Posterior Space

**Fig. 4:**
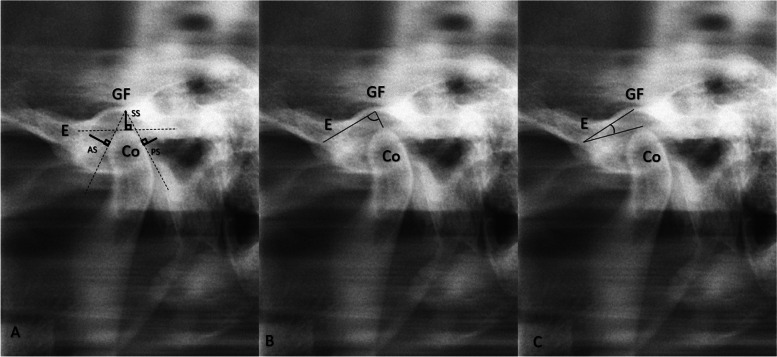
TMJ measurements in LPG. **A** Distance measurements **B**. Antero-posterior Angle (APA) **C**. Supero-inferior Angle (SIA). Co: Condyle, E: Articular eminence, GF: Glenoid Fossa, AS: Anterior Space, SS: Superior Space, PS: Posterior Space

The RadiAnt DICOM Viewer (version 4.6.9. (64-bit), Medixant, Poznan, Poland) was utilized to conduct MRI measurements in the mouth-closed position, T1 sequence, and all measurements were consistently taken at identical cross-sections, as illustrated in Fig. 2. Measurements of OPT and LPG were conducted utilizing the Turcasoft developed by Turcasoft, Samsun, Turkey). The results of these measurements are presented in Figs. 3 and 4.

The measurement of SS was obtained in linear units by determining the perpendicular distance from the tangent passing through the highest point of the condyle to the glenoid fossa. The AS refers to the perpendicular distance between the glenoid fossa and the tangent that intersects the point where the SS contacts the glenoid fossa and the most protuberant anterior point of the condyle. PS is defined as the vertical distance between the glenoid fossa and the tangent line that intersects the point of contact between the SS and the glenoid fossa and the most prominent posterior point of the condyle, according to sources [10, 15].

The present study involved the determination of two distinct angle measurements, namely the APA and the SIA. Specifically, the APA was defined as the angle formed between the apex of the articular eminence, the deepest point of the glenoid fossa, and the highest point of the condyle. On the other hand, the SIA was defined as the angle formed between the deepest point of the glenoid fossa, the apex of the eminence, and the highest point of the condyle.

The measurement discrepancies were utilized to simplify intricate measurements into a single value, and the decision tree was designed to be as uncomplicated and feasible as feasible. The obtained results involved the calculation of discrepancies in linear and angular measurements. This was achieved by computing the difference between the values of the affected side and the healthy side in the patient group and by determining the difference between the larger and smaller values in the control group. A singular value was obtained for each length and angle measurement in an individual patient based on the difference acquired. Consequently, a decision tree with a singular value was generated.

The study examined the measurement differences between the control and study groups and evaluated the significance level of these differences in patients with ADDwR and ADDwoR in the study group using the independent t-test method with a confidence level of 95%. The statistical software program utilized for data analysis in this study was IBM SPSS Version 25.0, developed by IBM and SPSS Inc. in Chicago, USA.

The IBM SPSS C5.0 algorithm was utilized to develop five distinct decision trees for the diagnosis of unilateral ADD based on measurement variations obtained from MRI, OPT, and LPG. The study was conducted by IBM and SPSS Inc. in Chicago, USA. The following decision trees are presented:

- A diagnostic decision tree was derived solely from differences in MRI measurements.
- The diagnostic decision tree was derived exclusively from differences in OPT measurements.
- The diagnostic decision tree was derived solely from differences in LPG measurements.
- The present study involves the generation of a diagnostic decision tree through the utilization of differences in OPT and LPG measurements.
- A diagnostic decision tree was derived based on discrepancies in measurements obtained through MRI, OPT, and LPG.

## Results

The research cohort comprised 89 individuals, comprising 21 males and 68 females, with an average age of 30.58 ± 12.01. Out of the total sample size of 89 patients, a majority of 60.7% (*n* = 54) were diagnosed with ADDwR, while the remaining 39.3% (*n* = 35) were diagnosed with ADDwoR. The study's control group comprised 72 participants, consisting of 20 males and 52 females, with a mean age of 31.75 ± 10.88.

A total of 161 LPG, OPT, and MRI scans were randomly selected and reevaluated by the same radiologist after a month after the initial measurement to assess the presence of measurement errors. The intraclass correlation coefficient (ICC) was utilized to evaluate intra-observer agreement in the analysis of values. The reliability of a measure can be assessed based on the ICC value. A value less than 0.5 indicates poor reliability, while a value between 0.5 and 0.75 suggests moderate reliability. A value between 0.75 and 0.9 indicates good reliability, and a value greater than 0.90 suggests excellent reliability. The study found that the reliability of the MRG, OPT, and LPG values was good, with agreement between the first and second measurements achieving coefficients of 0.86, 0.76, and 0.80, respectively [16].

An independent t-test was conducted to analyze the measurement discrepancies between patients with ADDwR and ADDwoR in the study cohort. The statistical analysis revealed significant differences in the angle measurements conducted in LPG. However, the linear measurements did not exhibit any statistically significant difference, as presented in Table 1.

**Table 1:** Independent t-test results of measurement differences between ADDwR and ADDwoR patients within the study group

**Imaging modalities**	**Measurement**	**ADDwR** **Mean ± SD**	**ADDwoR** **Mean ± SD**	***p* value**
MRI	APA Difference	7.16 ± 10.85	7.65 ± 10.15	***.834***
	SIA Difference	-1.33 ± 6.51	-2.69 ± 7.99	***.382***
	AS Difference	0.50 ± 0.81	0.34 ± 0.97	***.402***
	SS Difference	-0.19 ± 0.86	-0.40 ± 0.95	***.295***
	PS Difference	-0.03 ± 0.78	-0.05 ± 0.76	***.887***
OPT	APA Difference	2.16 ± 15.10	-3.36 ± 18.45	***.138***
	SIA Difference	-2.71 ± 13.28	-3.94 ± 12.38	***.675***
	AS Difference	-0.09 ± 0.88	-0.09 ± 0.76	***.985***
	SS Difference	2.54 ± 2.64	2.11 ± 3.01	***.498***
	PS Difference	-0.65 ± 2.81	0.24 ± 2.62	***.149***
LPG	APA Difference	2.60 ± 18.72	-8.45 ± 21.04	.012*
	SIA Difference	0.36 ± 6.90	-2.80 ± 5.18	.024*
	AS Difference	0.22 ± 1.26	-0.04 ± 0.81	***.230***
	SS Difference	2.11 ± 2.05	1.97 ± 2.52	***.776***
	PS Difference	0.09 ± 1.30	0.28 ± 1.64	***.557***

*Abbreviations*: *ADDwR* Anterior Disc Displacement with Reduction, *ADDwoR* Anterior Disc Displacement without Reduction, *MRI* Magnetic resonance imaging, *OPT* Orthopantomographic, *LPG* Lateral Panaromic Graphy, *APA* Antero-Poserior Angle, *SIA* Supero-inferior Angle, *AS* Anterior Space, *SS* Superior Space, *PS* Posterior Space

^*^Independent t test, statistically significant difference within 95% confidence limits (*p* < 0.05)

The study group's measurement differences in MRI, OPT, and LPG were subjected to a statistically independent t-test in comparison to the measurement differences of the control group's patients. With the exception of the AS discrepancy in MRI, all other variations in measurements were determined to be statistically significant, as presented in Table 2. The findings indicate that the study group patients exhibited significant increases in APA and significant decreases in SIA, SS, and PS as measured by MRI. The study conducted on OPT and LPG revealed that AS and APA exhibited a significant increase, while SIA, SS, and PS demonstrated a significant decrease. Therefore, the null hypothesis was rejected.

**Table 2:** Independent t-test results of measurement differences between control group and study group

**Imaging modalities**	**Measurement**	**Control Group Mean ± SD**	**Study Group** **Mean ± SD**	***p* value**
MRI	APA Difference	2.58 ± 2.25	7.23 ± 10.81	.000*
	SIA Difference	2.77 ± 2.41	-1.86 ± 7.12	.000*
	AS Difference	0.49 ± 0.36	0.44 ± 0.87	***.613***
	SS Difference	0.61 ± 0.45	-0.27 ± 0.90	.000*
	PS Difference	0.44 ± 0.35	-0.04 ± 0.77	.000*
OPT	APA Difference	11.83 ± 10.62	-0.002 ± 16.89	.000*
	SIA Difference	9.86 ± 8.29	-3.78 ± 15.47	.000*
	AS Difference	0.67 ± 0.57	-0.13 ± 1.96	.000*
	SS Difference	1.29 ± 0.99	2.34 ± 2.78	.002*
	PS Difference	1.81 ± 1.61	0.142 ± 5.03	.000*
LPG	APA Difference	13.48 ± 9.21	-3.03 ± 20.19	.000*
	SIA Difference	4.90 ± 4.33	-1.36 ± 7.54	.000*
	AS Difference	0.85 ± 0.92	0.11 ± 1.10	.000*
	SS Difference	0.91 ± 0.66	2.05 ± 2.24	.000*
	PS Difference	1.14 ± 1.15	0.16 ± 1.44	.000*

*Abbreviations*: *MRI* Magnetic resonance imaging, *OPT* Orthopantomographic, *LPG* Lateral Panaromic Graphy, *APA* Antero-Poserior angle, *SIA* Supero-inferior angle, *AS* Anterior Space, *SS* Superior Space, *PS* Posterior Space

^*^Independent t test, statistically significant difference within 95% confidence limits (*p* < 0.05)

### A diagnostic decision tree that solely utilizes differences in MRI measurements

**Fig. 5:**
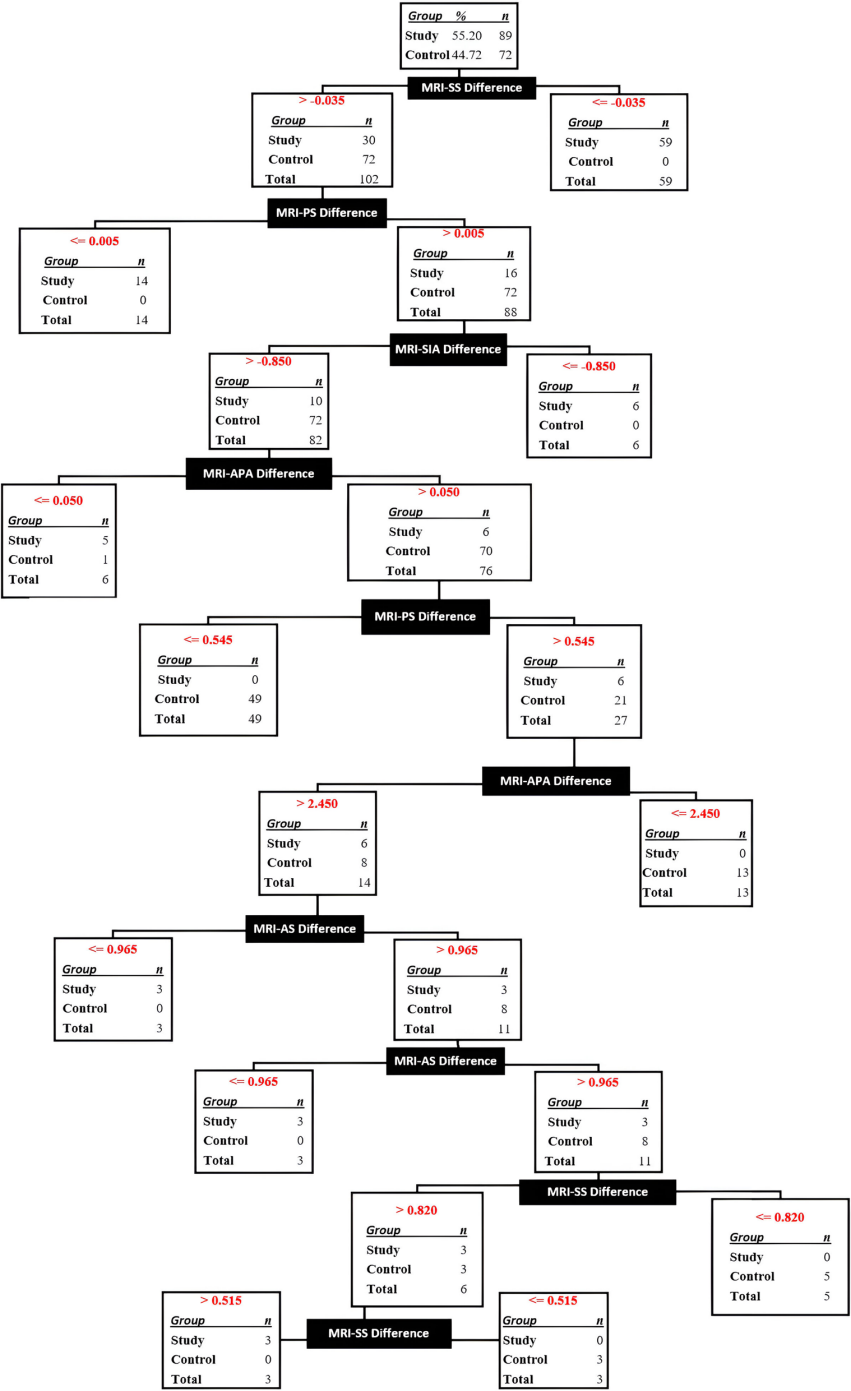
Decision tree created by SPSS algorithm using measurement differences on MRI. MRI: Magnetic resonance imaging, APA: Antero-Posterior Angle, SIA: Supero-inferior Angle AS: Anterior Space, SS: Superior Space, PS: Posterior Space

The decision tree constructed solely based on the measurement differences acquired from MRI revealed that out of the total of 161 patients in both the control and study groups, only one patient (0.62%) was misclassified. The remaining 160 patients (99.38%) were accurately distinguished as either having ADD or normal. The misclassification took place during the fourth stage, as depicted in Fig. 5.

### The diagnostic decision tree that solely utilizes differences in OPT measurements

**Fig. 6:**
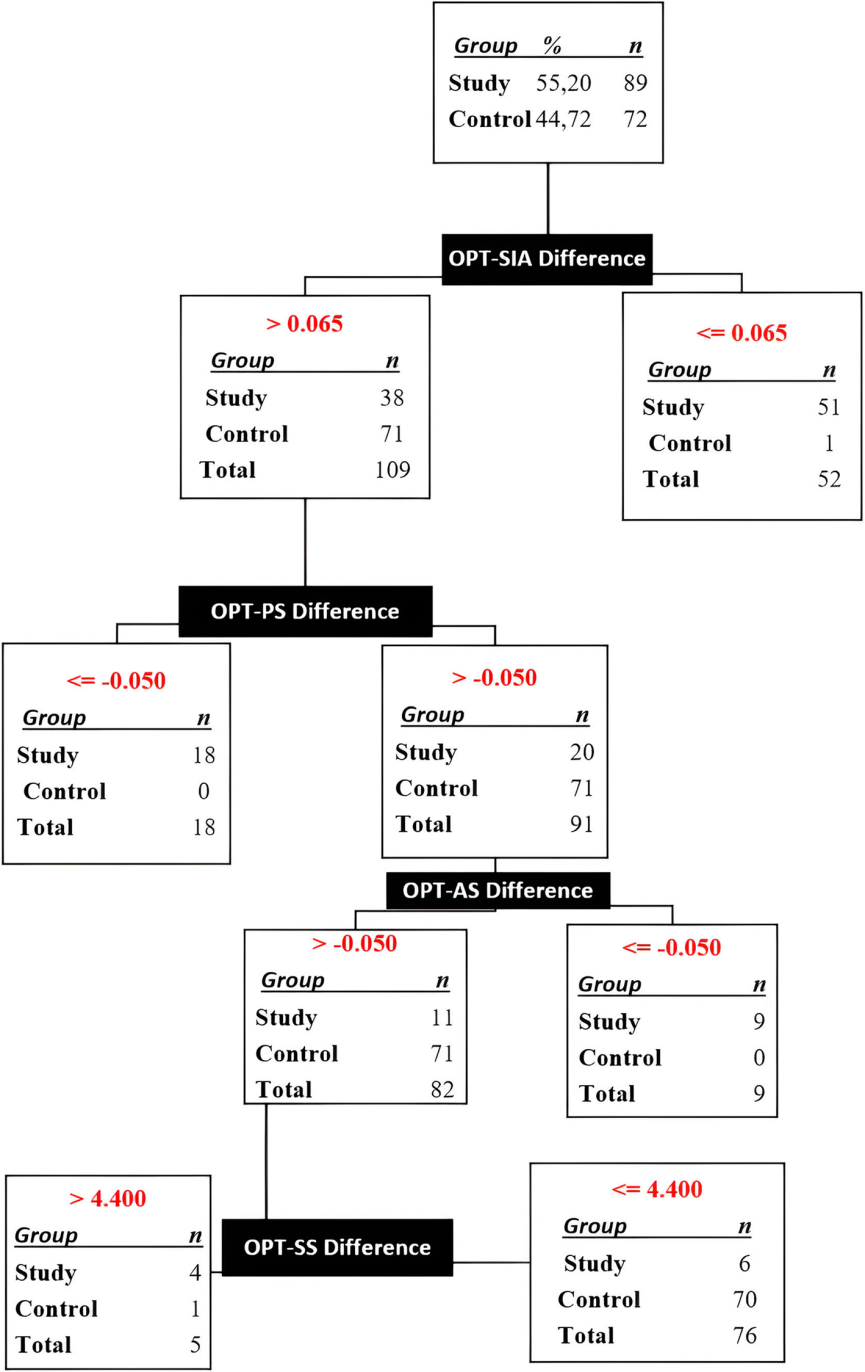
Decision tree created by SPSS algorithm using measurement differences on OPT. OPT: Orthopantomographic, APA: Antero-Posterior Angle, SIA: Supero-inferior Angle AS: Anterior Space, SS: Superior Space, PS: Posterior Space

The decision tree was constructed based on the measurement differences derived exclusively from OPT. The analysis revealed that out of the total of 161 patients in the control and study groups, 8 individuals (4.96%) were erroneously classified. Additionally, one participant (0.62%) had a missing value at step 4, while the remaining 152 (94.42%) were accurately distinguished as either having ADD or being in good health. The first and fourth steps (as shown in Fig. 6) were subject to erroneous categorization.

### The diagnostic decision tree was derived solely from the differences in LPG measurements

**Fig. 7:**
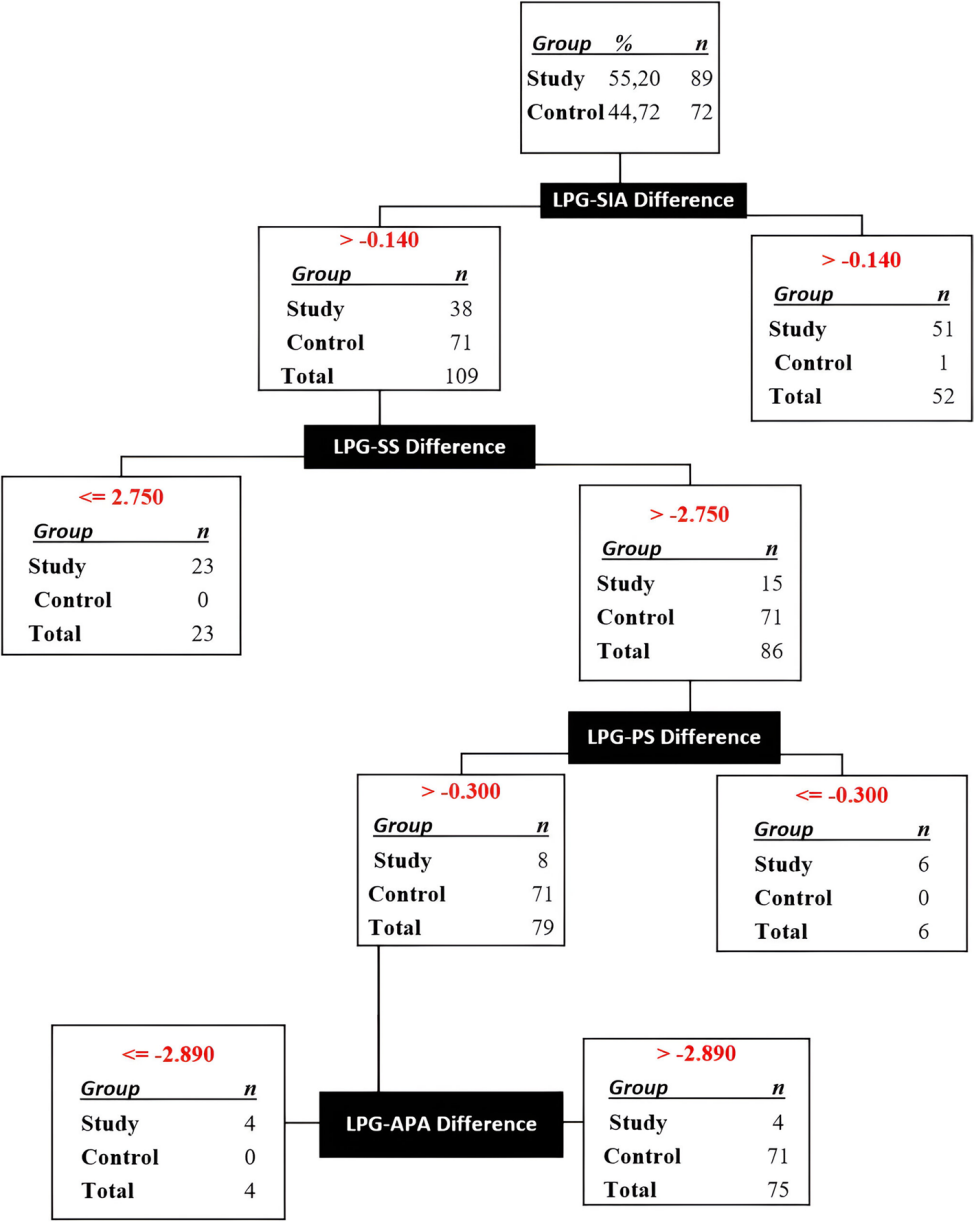
Decision tree created by SPSS algorithm using measurement differences on LPG. LPG: Lateral Panaromic Graphy, APA: Antero-Poserior Angle, SIA: Supero-inferior Angle, AS: Anterior Space, SS: Superior Space, PS: Posterior Space

The decision tree constructed solely based on the measurement differences derived from LPG resulted in the misclassification of 5 patients (3.1%) from the control and study groups. Additionally, one missing value (0.62%) was observed at step 3. However, the remaining 155 patients (96.28%) were accurately classified as either ADD or healthy, and this was achieved through a four-step grouping process. The first and fourth steps (as illustrated in Fig. 7) were subject to erroneous categorization.

### The present study concerns the development of a diagnostic decision tree based on the differences in OPT and LPG measurements

**Fig. 8:**
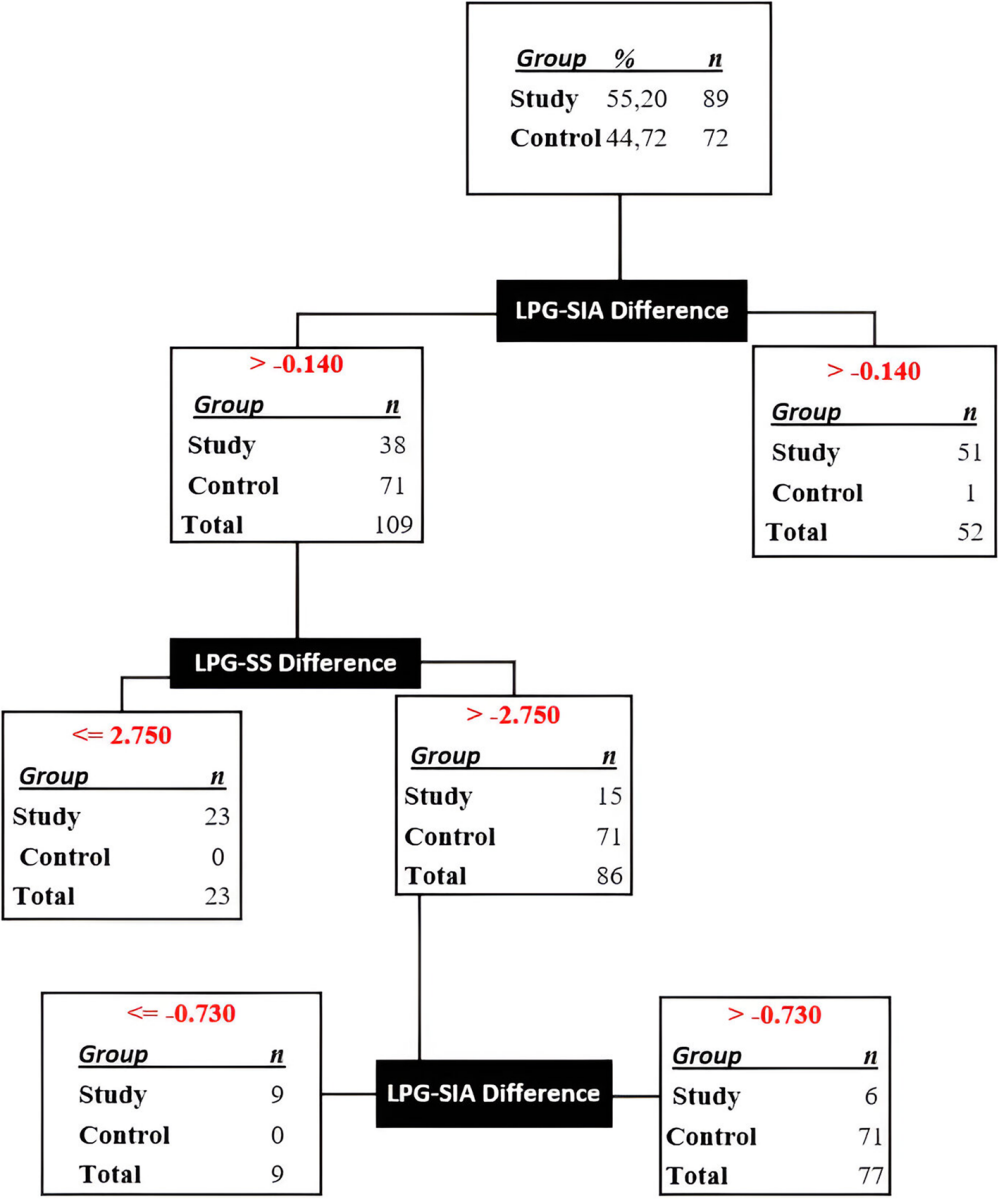
Decision tree created by SPSS algorithm using measurement differences on OPT and LPG. OPT: Orthopantomographic, LPG: Lateral Panoramic Graphy, APA: Antero-Posterior Angle, SIA: Supero-inferior Angle, AS: Anterior Space, SS: Superior Space, PS: Posterior Space

The decision tree, which was constructed based on the measurement differences derived from OPT and LPG, resulted in the misclassification of 7 patients (4.35%) out of a total of 161 patients in both the control and study groups. However, the remaining 154 patients (95.65%) were accurately classified as either having ADD or normal using a three-step process. The first and third steps (Fig. 8) were subject to erroneous categorization. Also, while categorizing in this decision tree, the algorithm has not used the OPT measurements.

### A diagnostic decision tree was derived utilizing discrepancies in measurements obtained with MRI, OPT and LPG

**Fig. 9:**
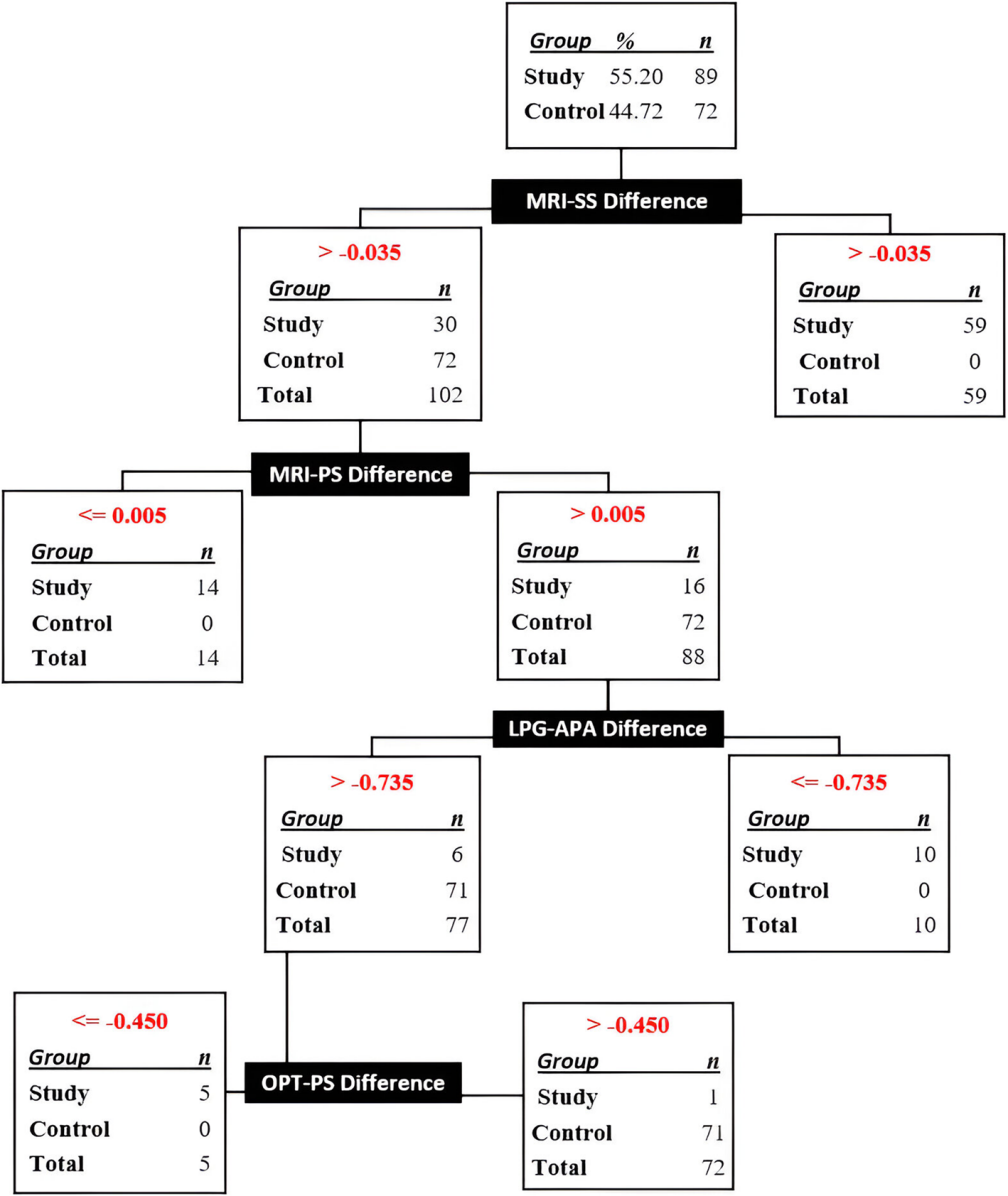
Decision tree created by SPSS algorithm using measurement differences on MRI, OPT and LPG. MRI: Magnetic resonance imaging, OPT: Orthopantomographic, LPG: Lateral Panoramic Graphy, APA: Antero-Posterior Angle, SIA: Supero-inferior Angle, AS: Anterior Space, SS: Superior Space, PS: Posterior Space

The decision tree constructed solely based on the measurement differences obtained from MRI, OPT, and LPG resulted in the misclassification of 1 (0.62%) out of 161 patients in both the control and study groups. However, the remaining 160 patients (99.38%) were accurately distinguished as either having ADD or normal. The misclassification took place during the second stage, as illustrated in Fig. 9.

## Discussion

The objective of this investigation is to diagnose unilateral ADD through the utilization of condyle position alterations in patients undergoing OPT and LPG without the requirement for MRI. In this study, decision trees were constructed based on three variations in length and two variations in angle measurements. The categorization of high-accuracy unilateral ADD was conducted using OPT and/or LPG, with and without MRI.

The present investigation revealed that the age and gender distributions of the control and study groups were comparable and exhibited a high degree of similarity. The study group and control group exhibited comparable age and gender distributions, thereby reducing the potential impact of age and gender-related factors on the outcomes.

According to Lee et al. [17], the reliability of utilizing MRI for monitoring bone changes is limited. According to previous research, CBCT has been identified as the most effective approach for assessing bone structure [18, 19]. The literature indicates that MRI images are capable of tracking the external boundaries of bones and analysing bone alterations and dimensions [8, 20–23]. The utilization of measurement discrepancies between the TMJ affected by ADD and the unaffected TMJ in healthy individuals was motivated, in part, by the contentious viewpoint regarding bone measurement in MRI.

Research findings indicate that patients with ADD exhibit posterior displacement and reduced length of the condyle [13, 15, 24–27]. According to Zhuo et al. [28], the condyle on the affected side of individuals with ADD exhibited a decrease or cessation in bone formation. Studies have reported that patients with ADD experience an increase in as well as a decrease in SS and PS based on joint space measurements [10–12, 14, 15, 25, 29, 30]. In the context of ADDwoR, it was observed that the SS of affected joints was smaller than that of healthy joints, with a substantial difference in measurements. Moreover, significant variations were noted in both SS and PS measurements between ADDwoR and ADDwoR, as reported in previous studies [11, 13, 29, 30]. According to Seo et al. [26], the reduction in condyle depth and height has been identified as the underlying cause. The initial step in decision trees that employ MRI-derived measurement differences involves the appearance of SS as the primary determinant. In decision trees that utilize OPT and/or LPT measurement differences, the initial step involves the occurrence of the SIA angle measurement difference. However, the initial stage involves the acquisition of SS via MRI in the decision-making process, wherein the variations in measurements obtained from all three methodologies are considered. Within this particular context, it is asserted that the superior movement holds a preeminent position in ADD pathologies and plays a crucial role in the disease's diagnostic process.

Within the literature, individuals with ADD have been juxtaposed with those who have ADD and unimpaired joints. The degree of severity of these changes is more pronounced in individuals with advanced ADD, as evidenced by the posterior displacement of the mandible, reduction in condyle height, asymmetrical joint growth and bone changes, and the progressive increase in SS reduction as the ADD disease advances [24, 31–33]. The study conducted by De Pontes et al. [13] utilizing MRI revealed significant joint space changes in both healthy individuals and those diagnosed with ADD. However, the authors noted that there was no statistically significant difference observed between individuals with ADD with and without reduction (ADDwR and ADDwoR, respectively). The study revealed a significant statistical disparity in the measurements of healthy individuals and those diagnosed with ADD. However, no significant difference was detected between ADD patients with and without reduction within the study group. The study did not incorporate individuals with advanced ADD who have undergone bone replacement procedures. Consequently, there was no statistically significant distinction observed in the measurements obtained from patients diagnosed with ADD with and without the presence of hyperactivity.

The study involved a comparison between patients exhibiting unilateral ADD and a control group. The statistical analysis revealed a significant difference in the mean measurement values of SS, PS, and SIA in MRI, with the former group exhibiting lower values. Consequently, the study group exhibited a decrease in SS, PS, and SIA. This study found a statistically significant increase in the APA angle among participants, as evidenced by a higher mean of the measurement differences of APA in MRI. The patient's superior and posterior synovial sheaths narrow within the joint among individuals with unilateral adduction. The observed constriction in both SS and PS is indicative of posterior and superior displacement of the condyle, which is consistent with existing literature. Furthermore, with regard to angle measurements, it is noteworthy that only the condyle is capable of movement, as two out of the three points remain stationary. Hence, the condyle is the sole determinant of the alterations in the angles. A reduction in the SIA measurement discrepancy implies an upward movement of the condyle, whereas an elevation in the APA measurement discrepancy suggests a posterior movement of the condyle.

The aetiology of the augmented prevalence of AS is hypothesized to be attributed to the hypertrophy of the posterior band of the TMJ disc and the anterior displacement of the disc in individuals diagnosed with ADD. To reduce the potential confounding effects of variations in measurements, this study excluded cases with altered bone morphology from our study. As a result, our findings did not reveal a statistically significant difference in the assessment of AS using MRI.

According to Mupparapu et al. [34], OPT presents several advantages as an imaging technique, including high resolution and specificity, ease of access, and low cost. Nevertheless, it is widely acknowledged that OPT exhibits suboptimal sensitivity and accuracy levels [3]. The OPT is commonly utilized and offers comprehensive insights pertaining to the TMJ. Despite some opposing views regarding the use of OPT as the primary diagnostic tool for TMJ, it has been suggested that OPT can be utilized for TMJ evaluations provided that the examiner possesses the requisite expertise [3, 8]. According to the study conducted by Gilboa et al. [35], the OPT imaging technique was found to be effective in visualizing the articular eminence and glenoid fossa, and their precise locations could be reliably determined. According to Fallon et al. [36], the positioning of the eminence and glenoid fossa can be altered due to an incorrect angle of the X-ray. According to the study conducted by Helenius et al. [37], MRI provides the most effective visualization of the TMJ space, and it is not recommended to conduct joint space measurements using OPT-LPG. The study did not employ OPT and LPG for joint space measurements but rather relied on the observation of changes in distance and angles using specific fixed points. The success rate of categorization was comparatively lower in OPT and LPG as opposed to MRI due to the aforementioned reasons. The study revealed statistically significant differences in the measurements of OPT between the groups assigned to the experimental and control conditions. Consequently, despite the argument against the utilization of OPT in diagnosis, the statistical significance of the measurements indicates that the ailment can be detected through OPT.

LPG employs a reduced amount of radiation in contrast to OPT [34]. In the context of TMJ imaging, it has been observed that in LPG, the rays are projected at a more perpendicular angle to the long axis of the condyle as compared to OPT. It has been suggested that OPT employs a more appropriate angle for diagnostic purposes, as supported by previous literature [34, 38]. According to Beloor Vasudeva et al. [3], the evaluation of the glenoid fossa and condyle in LPG was found to be more distinct compared to that of OPT. According to reports, the utilization of LPG could prove advantageous in the assessment of TMJ, as it allows for the acquisition of images with the patient's oral cavity in an open position. The rationale for positioning the patient with an open mouth is to prevent any potential bone superposition that may arise. Pullinger and Seligman [12] have suggested that the accuracy of TMJ space measurements can be improved by utilizing joint images captured in the closed-mouth position. The present investigation involved the acquisition of closed-mouth position images through the use of LPG and MRI, which were subsequently subjected to measurement. According to studies in literature, the optimal method for conducting a TMJ examination via MRI involves utilizing sagittal and oblique sagittal sections. In our investigation, the MRI measurements were conducted using oblique sagittal sections with closed-mouth positioning [39, 40]. Positioning errors in OPT and LPG may result in excessive magnification and distortion. The study excluded positioning errors due to this rationale. Rather than utilizing the measurement outcomes in a direct manner, the alteration in the measurement was observed by means of the disparities in the measurements. Decision trees were devised to account for these variations in measurements. Furthermore, the ICC values obtained from the magnification and distortions in OPT and LPG in the study were comparatively lower than those of MRI.

The results of the LPG measurement analysis indicated a statistically significant disparity between the experimental and control cohorts. Consequently, the measurements conducted in LPG have the potential to aid in the diagnosis of ADD. Furthermore, LPG measurements were utilized throughout all stages of the decision tree that was constructed based on the measurements acquired from OPT and LPG. This indicates that the efficacy of measurements conducted in LPG surpasses that of OPT.

The study findings indicate a noteworthy reduction in the measurement discrepancies of SS, PS, and SIA and a significant elevation in the measurement discrepancies of AS and APA when comparing the study group with the patient group in OPT and LPG. This research demonstrates that unilateral ADD patients exhibit a significant and observable alteration in the condyle in OPT and LPG, in addition to MRI findings. Furthermore, it has been noted that while the difference in AS measurement is not significant in MRI, it is significant in OPT and LPG. The observed dissimilarity between the experimental and control cohorts in the variations detected across all three methodologies indicates that there is a quantifiable alteration in unilateral ADD patients when utilizing any of the aforementioned techniques.

According to the study, the evaluation of TMJ structures is limited to the condyles due to the oblique angle of the rays in an OPT in relation to the long axis of the condyle. According to the aforementioned study, it has been reported that approximately 60–70% of alterations in the bone can be observed, with the condyle exhibiting a broader appearance in both OPT and LPG imaging modalities. Previous literature has indicated that OPT has the capability to image a substantial area and that examination of the TMJ can be conducted through the use of OPT or LPG [4, 41–43]. According to another study, OPT exhibits both vertical and horizontal magnifications, leading to greater uncertainty in horizontal measurements as compared to vertical ones and alterations in the horizontal dimension of LPG have an impact on the placement of the condyles. Nevertheless, the data acquired in this investigation did not exhibit statistical significance [3]. Kambylafkas et al. [44] highlighted the appropriateness of OPT for assessing vertical asymmetry.

A comparative analysis of decision tree studies encompassing all three imaging modalities is absent from the existing literature. The observed dissimilarity in the measurement variances between the study and control cohorts across all three methodologies can serve as substantiation for the manifestation of ADD as an alteration in bone positioning. Furthermore, the reduced number of decision tree steps in the absence of MRI measurements suggests that the osteoarticular changes associated with ADD pathology can be more distinctly visualized through OPT and LPG imaging modalities. Despite the relatively low number of steps in the decision trees that did not incorporate MRI, the accuracy of correct categorization could not be established with the same level of precision as that observed in the decision trees that utilized MRI. Nonetheless, it is worth noting that the decision trees lacking MRI exhibited a success rate of correct categorization that did not fall below 95% in any instance.

The absence of soft tissue assessment capabilities in OPT and LPG imaging modalities, in contrast to MRI, may limit the ability to evaluate pathologies affecting both soft and hard tissues. In certain settings where resources are constrained, OPT and LPG may serve as initial diagnostic tools to optimize the utilization of MRI and facilitate the assessment of the necessity for an MRI diagnosis.

Potential limitations of this study is its retrospective nature, which restricted our evaluation to radiological data and precluded access to clinical observations. Furthermore, ADD may manifest as a standalone condition in certain instances; however, it may also be accompanied by additional symptoms and observations. Hence, the limitation of our study lies in the fact that it was conducted solely on unilateral ADD patients in isolation. Future studies should investigate the validity of this method by employing large sample sizes, utilizing a prospective design, conducting clinical examinations, and including groups with more complex ADD presentations.

## Conclusions

Observation of changes in condyle position can be achieved through the utilization of measurement differences in patients with unilateral ADD, as evidenced by OPT and LPG.

There was no observable displacement of the condyle that could be considered significant between the imaging modalities of ADDwR and ADDwoR. This was found to be the case across all three modalities.

Decision trees constructed based on differences in linear and angular measurements can achieve a diagnostic accuracy of over 94% for unilateral ADD in the OPT and/or LPG.

## Data Availability

The datasets used and/or analysed during the current study are available from the corresponding author on reasonable request.
